# Qualitative and quantitative evaluation of phosphate-solubilizing ability of *Lysinibacillus macroides*: a climate-resilient biofertilizer candidate for sustainable crop nutrition

**DOI:** 10.3389/fmicb.2026.1849362

**Published:** 2026-06-02

**Authors:** Pushpa Gehlot, Jyoti Yadav, Tripta Jain

**Affiliations:** Department of Botany, Mohanlal Sukhadia University, Udaipur, Rajasthan

**Keywords:** *Lysinibacillus macrolides*, phosphate-solubilizing bacteria, rhizosphere microbiome, seed biopriming, sustainable phosphorus management

## Abstract

Phosphorus (P) is a critical macronutrient governing plant productivity, however its bioavailability in agricultural soils remains severely constrained due to fixation in insoluble mineral complexes, particularly calcium-bound phosphates in alkaline systems. The low use efficiency of chemical fertilizers, coupled with escalating environmental concerns, necessitates biologically driven strategies for sustainable phosphorus management. In this study, rhizospheric bacteria associated with chili (*Capsicum annuum* L.) were systematically investigated to elucidate the phosphate-solubilizing potential of *Lysinibacillus macroides*, an underexplored spore-forming plant growth-promoting rhizobacterium (PGPR). A total of 23 isolates were characterized using integrated qualitative and quantitative assays. All isolates exhibited halo formation on Pikovskaya’s agar (1.26–4.70 mm), while NBRIP broth analysis revealed substantial tricalcium phosphate solubilization, reaching up to 30.03 μg mL^–1^. This activity was consistently associated with pronounced acidification, with pH declining from 7.0 to 3.2–4.1, indicating an acidification-driven solubilization mechanism. A strong inverse correlation between soluble phosphate and pH (*r* = −0.91, *p* < 0.01), supported by multivariate analysis, suggests that phosphate mobilization is governed by metabolically regulated acidification dynamics, likely mediated through organic acid production and proton extrusion. Among the isolates, 2.B (*L. macroides*) and 4.1 (*L. fusiformis*) exhibited superior solubilization efficiency and were distinctly separated through clustering analysis. Functional validation through seed biopriming demonstrated significant enhancement in germination (96.66%) and Seedling Vigor Index (766.88), establishing a direct linkage between microbial phosphorus mobilization and early plant development. *Lysinibacillus macroides* maintained significant growth across a broad range of pH (4–10), temperature (15–45°C), and salinity (0.01–0.1% NaCl), demonstrating its inherent tolerance to multiple abiotic stresses and confirming its potential as a climate-resilient plant growth-promoting bacterium. This study provides a quantitative and multivariate demonstration of acidification-coupled phosphate solubilization in *Lysinibacillus macroides*, integrating biochemical, statistical, and plant-based validation to elucidate a consistent mechanism of phosphorus mobilization. Collectively, the findings establish *L. macroides* as a resilient biofertilizer candidate for enhancing phosphorus-use efficiency and sustainable nutrient management in chili and other crops.

## 1 Introduction

Phosphorus (P) is a fundamental macronutrient governing plant growth, productivity, and metabolic regulation. As a structural component of nucleic acids, phospholipids, and ATP, phosphorus drives photosynthesis, respiration, signal transduction, and energy transfer processes that sustain plant development (Bechtaoui et al., 2021). Despite its abundance in soils, phosphorus remains one of the most limiting nutrients in agricultural systems because its bioavailability is tightly constrained by geochemical immobilization (Khan et al., 2023). Less than 0.1% of total soil phosphorus exists in plant-accessible orthophosphate forms (H_2_PO_2_^–^ and HPO^42–^), while the majority is sequestered in insoluble complexes with calcium in alkaline soils and with iron and aluminum oxides in acidic soils (Shi et al., 2023). This physicochemical fixation severely restricts plant uptake, thereby limiting crop productivity even in P-rich soils (Bafiec and Bangady Killur, 2022). To offset this limitation, modern agriculture relies heavily on chemical phosphate fertilizers derived from finite phosphate rock reserves. However, fertilizer use efficiency rarely exceeds 20–25%, as applied phosphorus rapidly precipitates or becomes sorbed onto soil particles, re-entering non-labile pools (Kataria et al., 2024). The over-application of phosphate fertilizers not only increases production costs but also contributes to soil nutrient imbalance, groundwater contamination, and eutrophication of freshwater ecosystems (Alom et al., 2025). With global phosphate rock resources declining and fertilizer prices escalating, improving phosphorus use efficiency has emerged as a critical priority for sustainable agriculture and environmental stewardship (Adnan et al., 2025).

Biological mobilization of soil phosphorus through rhizospheric microorganisms represents a promising eco-friendly alternative to conventional fertilization strategies. Plant growth-promoting rhizobacteria (PGPR) enhance plant nutrition through multiple mechanisms, including nitrogen fixation, phytohormone production, siderophore secretion, and stress alleviation (Gehlot et al., 2025; Sun et al., 2024). Within this functional group, phosphate-solubilizing microorganisms (PSM) play a central role in the cycling of mineral phosphorus. These organisms convert insoluble inorganic phosphates into soluble forms primarily by secreting low-molecular-weight organic acids, extruding protons, and hydrolyzing them enzymatically. Organic acids such as gluconic, citric, oxalic, and lactic acids chelate cations (Ca^2 +^, Fe^3 +^, Al^3 +^) and lower rhizospheric pH, thereby enhancing mineral dissolution and orthophosphate release (Luo et al., 2025). Consequently, PSMs act as biocatalysts of soil mineral weathering and contribute to improved phosphorus bioavailability at the root-soil interface (Zhu et al., 2024).

Extensive research has focused on phosphate solubilization by genera such as *Bacillus*, *Pseudomonas*, *Rhizobium*, and *Trichoderma*, which have been widely investigated for biofertilizer development (Sharma et al., 2025). Despite extensive characterization, many of these well-studied taxa exhibit inconsistent field performance due to environmental sensitivity, limited persistence under abiotic stress, and variable phosphate-solubilization efficiency across soil types. Furthermore, a large proportion of studies remain confined to laboratory-scale screening, with insufficient integration of quantitative solubilization dynamics, mechanistic validation, and plant-level performance. This creates a critical gap in translating promising PSM candidates into reliable biofertilizer formulations. In addition, current literature predominantly emphasizes a narrow range of model genera, resulting in an underrepresentation of potentially robust and stress-resilient taxa capable of functioning under challenging agroecological conditions such as semi-arid and alkaline soils. In addition, current literature predominantly emphasizes a narrow range of model genera, resulting in an underrepresentation of potentially robust and stress-resilient taxa capable of functioning under challenging agroecological conditions such as semi-arid and alkaline soils. In particular, the genus *Lysinibacillus* has received comparatively limited attention despite its frequent occurrence in rhizospheric environments and its documented resilience under environmental stress (Li et al., 2024). Members of this genus are Gram-positive, spore-forming bacteria characterized by robust survival capacity, metabolic versatility, and ecological adaptability. The ability to form endospores provides a significant advantage for inoculant formulation, long-term storage stability, and persistence under fluctuating temperature and moisture conditions (Dolatabad, 2026).

Among these species, *Lysinibacillus macroides* remains under-characterized with respect to phosphorus mobilization. Existing reports on *Lysinibacillus* spp. are fragmented and largely qualitative, with limited comparative data on strain-level variability, phosphate solubilization kinetics, and their relationship with environmental parameters such as pH dynamics. Moreover, there is a lack of systematic evaluation linking biochemical solubilization traits with functional agronomic outcomes, particularly under crop-specific rhizospheric conditions. Preliminary studies suggest that certain *Lysinibacillus* strains may participate in nutrient cycling; however, systematic and quantitative evaluation of their phosphate-solubilizing efficiency, solubilization dynamics, and strain-level variability is lacking (Leiva-Mora et al., 2026). Furthermore, mechanistic evidence linking phosphate release to medium acidification and metabolic activity in this species remains scarce. Expanding the taxonomic spectrum of characterized phosfate-solubilizing bacteria is essential to diversify the microbial resources available for sustainable phosphorus management (Saranraj et al., 2022).

Chili (*Capsicum annuum* L.) cultivation, particularly in semi-arid regions such as Rajasthan, frequently encounters phosphorus-limited soils characterized by high calcium content and reduced P mobility (Almuktar et al., 2021). Chili was specifically selected as the model crop in this study due to its high phosphorus demand during critical growth stages, particularly for root development, flowering, and fruit formation, which makes it highly sensitive to phosphorus deficiency. Moreover, chili is an economically important horticultural crop widely cultivated in semi-arid and arid regions of India, where soil phosphorus availability is inherently low due to calcium-mediated fixation (Das et al., 2025). Unlike several cereal crops that may exhibit relatively higher tolerance to nutrient limitations, chili shows pronounced yield reductions under phosphorus-deficient conditions, thereby serving as a sensitive and reliable indicator system for evaluating the efficiency of phosphate-solubilizing microorganisms. Additionally, the chili rhizosphere harbors diverse and dynamic microbial communities, making it a suitable ecological niche for isolating functionally efficient and stress-adapted phosphate-solubilizing bacteria (Gehlot et al., 2026). Identifying resilient rhizospheric bacteria capable of mobilizing insoluble calcium phosphates could significantly enhance nutrient availability and reduce chemical fertilizer dependence in such agroecosystems (Etesami and Adl, 2020). Therefore, exploring indigenous microbial communities associated with chili rhizosphere provides a rational strategy for isolating ecologically adapted and agronomically relevant phosphate-solubilizing strains (Rajguru et al., 2024). *Lysinibacillus macroides* has emerged as a promising climate-resilient plant growth-promoting bacterium due to its ability to tolerate multiple abiotic stresses. In the present study, the isolate sustained growth across a broad range of pH (4–10), temperature (15–45°C), and salinity (0.01–0.1% NaCl), with optimal growth observed at neutral pH (7) and moderate temperatures (25–35°C). Notably, it maintained appreciable growth under alkaline conditions, elevated temperatures, and increasing salinity, indicating strong physiological adaptability. Such multi-stress tolerance highlights its potential suitability for application in climate-stressed and variable agroecosystems.

A critical gap exists in the integrated evaluation of underexplored phosphate-solubilizing bacteria, particularly *Lysinibacillus macroides*, combining quantitative efficiency, mechanistic understanding, and plant-level validation. The objective of this study was to systematically evaluate the phosphate-solubilizing potential of *Lysinibacillus macroides* isolates from chili rhizosphere through an integrated qualitative, quantitative, mechanistic, and plant-based approach. Phosphate solubilization dynamics and their relationship with pH variation were investigated to elucidate strain-dependent variability and the underlying mechanism of mineral dissolution. Additionally, functional validation was performed through seed biopriming assays to assess the agronomic relevance of selected isolates. The study postulated that selected *L. macroides* isolates would exhibit efficient acidification-driven phosphate solubilization and possess potential applicability as biofertilizer candidates. To our knowledge, this study represents one of the first integrated investigations highlighting the phosphate-solubilizing potential and agronomic relevance of *Lysinibacillus macroides* from chili rhizosphere soils. The study aimed to evaluate strain-dependent phosphate mobilization and to explore the relationship between phosphate release and acidification-driven mineral dissolution in an underexplored spore-forming genus. Unlike previous studies primarily focused on conventional phosphate-solubilizing genera such as *Bacillus* and *Pseudomonas* (Abdelgalil et al., 2022), the present work expands the functional and taxonomic diversity of recognized phosphate-solubilizing microorganisms and provides a foundation for future mechanistic, genomic, and field-level validation studies for sustainable phosphorus management in agroecosystems.

## 2 Materials and methods

### 2.1 Sample collection

Rhizospheric soil samples were collected from chili (*Capsicum annuum* L.) fields in Udaipur district, Rajasthan, India (24°34’50.3”N, 73°42’07.7”E). The selected plants were healthy, 60–70 days old, and free of visible disease symptoms. Approximately 10–15 g of soil adhering to roots (5–15 cm depth) was collected aseptically using sterile spatulas. Samples were aseptically collected in sterile polyethene bags and immediately transported to the laboratory in insulated ice boxes under controlled temperature conditions to preserve their physicochemical and microbiological integrity before further processing and analysis (de Andrade et al., 2023).

### 2.2 Isolation of phosphate-solubilizing bacteria

Ten grams of soil sample was suspended in 90 mL of sterile water and serially diluted up to 10^–6^. One hundred microlitres of dilutions (10^–3^ to 10^–6^) were spread on nutrient agar (NA) plates and incubated at 37 ± 2°C for 48 h. Distinct colonies were picked, streaked repeatedly to obtain pure cultures and maintained on NA slants at 4°C for routine use. Long-term preservation was done by storing glycerol stocks (20%) at −4°C (Aneja et al., 2008).

### 2.3 Morphological and biochemical characterization

Colonies were examined for color, margin, elevation and consistency. Gram staining was performed to confirm identity as Gram-positive and rod-shaped. Cell morphology was studied under light microscope Lieca DM 2000 microscope (Lieca Microsystems, Germany) at 400X magnification. Standard biochemical assays included catalase, nitrate reduction, indole production, MR-VP test and carbohydrate fermentation using Hi-Carbohydrate kits (KB009, HiMedia Laboratories Pvt. Ltd., Mumbai, India) (Aneja, 2007).

### 2.4 Molecular confirmation

All isolates were initially subjected to morphological and biochemical characterization. Based on preliminary phosphate-solubilization performance, selected representative high-efficiency isolates were further subjected to molecular identification using 16S rRNA gene sequencing. Representative high-efficiency isolates (2.B and 4.1) were selected for molecular identification through 16S rRNA gene sequencing. Genomic DNA was extracted using the CTAB method. The 16S rRNA gene was amplified with universal primers 16S Forward (GGATGAGCCCGCGGCCTA) and 16S Reverse (CGGTGTGTACAAGGCCCGG). The 16S rRNA gene was amplified using 169 ng of genomic DNA in a 50 μL reaction containing 1 μL each of 10 pM forward and reverse primers, 4 μL dNTPs (2.5 mM each), 10 μL of 10xTaq buffer, 1 μL Taq DNA polymerase (3 U/μL), and nuclease-free water. PCR conditions included initial denaturation at 94°C for 3 min, followed by 30 cycles of 94°C for 1 min, 50°C for 1 min, and 72°C for 2 min, with a final extension at 72°C for 7 min. Amplicons were stored at 4°C for further analysis (Haro et al., 2021).

PCR products were purified and sequenced bidirectionally (Biokart, India). Sequence identification was carried out using the BLASTn algorithm against the NCBI GenBank databases. Phylogenetic trees were constructed with MEGA X software (neighbor-joining) (Version 10.2.6, Pennsylvania State University, United States). Sequences were deposited under accession numbers PX482185 and PX482183 for 2.B and 4.1, respectively.

### 2.5 Qualitative phosphate solubilization assay

Primary screening of isolates was conducted on Pikovskaya’s agar (PVK), prepared with the following composition (per liter): glucose, 10 g; Ca*3*(PO_4_)_2_ (TCP), 5 g; (NH_4_)_2_SO_4_, 0.5 g; NaCl, 0.2 g; MgSO_4_⋅7H_2_O, 0.1 g; KCl, 0.2 g; yeast extract, 0.5 g; MnSO_4_⋅H_2_O, 0.01 g; FeSO_4_⋅7H_2_O, 0.01 g; agar, 15 g; distilled water, 1,000 mL; final pH 7.0 ± 0.2 (Pikovskaya, 1948). Plates were spot-inoculated with overnight bacterial cultures and incubated at 37 ± 2°C for 7 days. Formation of a clear halo zone around colonies indicated phosphate solubilization. Colony diameter and halo diameter were measured in millimeters, and the Solubilization Index (SI) was calculated (Edi-Premono et al., 1996).


$$P\,S\,I\;\frac{C\,o\,l\,o\,n\,y\,d\,i\,a\,m\,e\,t\,e\,r+h\,a\,l\,o\,z\,o\,n\,e\,d\,i\,a\,m\,e\,t\,e\,r}{C\,o\,l\,o\,n\,y\,d\,i\,a\,m\,e\,t\,e\,r}$$

### 2.6 Quantitative phosphate solubilization and effect of increasing tricalcium phosphate concentration

Quantitative estimation of phosphate solubilization was performed using National Botanical Research Institute’s Phosphate (NBRIP) broth (per liter: glucose 10 g; Ca*3*(PO_4_)_2_ 5 g; MgCl_2_⋅6H_2_O 5 g; MgSO_4_⋅7H_2_O 0.25 g; KCl 0.2 g; (NH_4_)_2_SO_4_ 0.1 g; distilled water 1,000 mL; pH 7.0) as per the method of Nautiyal (1999). For the standard assay, 50 mL Erlenmeyer flasks containing 20 mL of NBRIP broth were inoculated with 1 mL of 24 h old bacterial culture (10^8^) CFU mL^–1^) and incubated at 37 ± 2°C under shaking conditions (120 rpm) for 21 days. Uninoculated broth served as the control. To evaluate the effect of substrate concentration on phosphate solubilization, NBRIP broth was amended with graded concentrations of tricalcium phosphate (TCP) at 0.05, 0.10, 0.20, 0.30, and 0.40 mg mL^–1^. Culture tubes containing 20 mL of the respective medium were inoculated with 1 mL of freshly prepared bacterial suspension (∼10^8^) CFU mL^–1^) and incubated at 37 ± 2°C under identical conditions. All treatments were maintained in triplicate, and uninoculated controls were included for each TCP concentration.

After incubation, cultures were centrifuged at 8,000 rpm for 10 min to obtain cell-free supernatants. Soluble phosphate in the supernatant was quantified using the molybdenum blue method (Crouch and Malmstadt, 1967; Ismail, 2023). Briefly, 1 mL of culture supernatant was mixed with 0.5 mL ammonium molybdate solution (2.5% prepared in 5N H_2_SO_4_) and 0.1 mL stannous chloride solution (10%). Following 10 min of color development, absorbance was recorded at 800 nm against an appropriate blank. A standard calibration curve was constructed using KH_2_PO_4_, and soluble phosphate concentration was expressed as μg mL^–1^ (Sharan et al., 2008).

### 2.7 pH measurement

The pH of culture supernatants was measured at each sampling point using a calibrated digital pH meter (Eutech Instruments, MRL, Udaipur). Correlation analysis was performed between soluble phosphate and pH values.

### 2.8 Assessment of abiotic stress tolerance for climate resilience in *Lysinibacillus macroides*

The tolerance of *Lysinibacillus macroides* to key abiotic stress factors was evaluated to determine its climate-resilient potential. Freshly grown cultures were inoculated into broth media and subjected to varying environmental conditions, including pH (4–10), temperature (15, 25, 35, 40, and 45°C), and salinity (0.01–0.1% NaCl, w/v). The pH of the medium was adjusted using 1 N HCl or 1 N NaOH before sterilization. All cultures were incubated under shaking conditions (200 rpm) at 28 ± 2°C, except for temperature treatments, where incubation was carried out at the respective temperatures. After 24–48 h of incubation, bacterial growth was quantified by measuring optical density at 600 nm (OD_600_) using a spectrophotometer (ELICO Model: SL159), with uninoculated media serving as the control. All experiments were performed in triplicate (*n* = 3), and results were expressed as mean ± standard deviation. The ability of the isolate to sustain growth across a wide range of pH, temperature, and salinity conditions was considered indicative of its adaptability to multiple abiotic stresses, supporting its classification as a climate-resilient plant growth-promoting bacterium.

### 2.9 Seed biopriming assay

A total of 23 rhizobacterial isolates, previously recovered from the chili rhizosphere and systematically characterized for their plant growth-promoting attributes, were selected and utilized for the seed biopriming assay. Each isolate was cultured individually in sterile Nutrient Broth and incubated at 37 ± 2°C for 24–48 h under shaking conditions (150 rpm) until the logarithmic growth phase was reached. The cultures were centrifuged at 8,000 rpm × 10 min, and the resulting bacterial pellets were washed twice with sterile distilled water. The cell density of each suspension was adjusted to approximately 10^8^) CFU mL^–1^ using spectrophotometric measurement at 600 nm.

Certified seeds of chili (*Capsicum annuum* L.) variety Pusa Jwala used in the experiment were purchased from a local agricultural seed shop in Udaipur, Rajasthan, India. Seeds were surface sterilized by immersion in 0.1% (w/v) mercuric chloride (HgCl_2_) solution for 2–3 min, followed by thorough rinsing at least five times with sterile distilled water to ensure complete removal of residual sterilant before further experimental use. The sterilized seeds were air-dried under aseptic conditions. For biopriming, seeds were immersed in freshly prepared bacterial suspensions (10^8^) CFU mL^–1^) for 6 h at room temperature. After incubation, seeds were removed and air-dried in a laminar airflow cabinet to restore their original moisture level. Seeds treated with sterile distilled water served as the uninoculated control (Abdul Mutalib et al., 2025).

Bioprimed seeds were placed in sterile 90 mm petri plates lined with double-layered Whatman No. 1 filter paper moistened with sterile distilled water. Each treatment consisted of 10 seeds per petri plate with three independent replicates arranged in a Completely Randomized Design (CRD). The plates were incubated in a growth chamber at 25 ± 2°C under a 12 h light/12 h dark photoperiod, and moisture was maintained by periodic addition of sterile distilled water as needed. After 14 days of incubation, germination percentage (based on radicle emergence), root length (cm) and shoot length (cm) were recorded. The Seedling Vigor Index (SVI) was calculated as Abdul-Baki and Anderson (1973).


Seed⁢vigor⁢index:germination%⁢X⁢seedling⁢length⁢(cm)

Where⁢Seedling⁢Length=RootLength+Shoot⁢Length.

### 2.10 Statistical analysis

All data were expressed as mean ± standard deviation (SD). Statistical differences among treatments were analyzed using one-way analysis of variance (ANOVA), followed by Tukey’s Honest Significant Difference (HSD) test at *p* ≤ 0.05. Statistical analyses were performed using OriginPro 2026 software (Version 10.3, OriginLab Corporation, Northampton, MA, United States).

To determine the relationship between phosphate solubilization efficiency and medium acidification, Pearson’s correlation analysis was performed using mean values of Solubilization Index (SI), quantitatively estimated soluble phosphate (μg mL^–1^), and final culture pH. Correlation coefficients (r) were computed using OriginPro 2026 (Version 10.3, OriginLab Corporation, Northampton, MA, United States). Statistical significance was evaluated at *p* < 0.05 and *p* < 0.01 levels (Yang et al., 2022). Pearson correlation matrix was constructed to evaluate the strength and direction of relationships among the measured variables. Correlation coefficients (r) were calculated to determine the degree of linear association between parameters. Positive values indicated direct relationships, whereas negative values represented inverse associations. The magnitude of the coefficients reflected the strength of the correlation, and statistical significance was determined at the appropriate probability level (*p* < 0.05).

## 3 Results

### 3.1 Morphological and biochemical characteristics

A total of 23 morphologically distinct bacterial isolates were obtained from the rhizosphere of *Capsicum annuum* L. The isolates exhibited considerable phenotypic diversity on nutrient agar, varying in colony color (cream, off-white, yellow), texture (smooth to rough), growth rate (slow to fast), and morphology. Gram staining revealed a predominance of Gram-positive rods, although Gram-negative rods and cocci were also present, indicating taxonomic heterogeneity within the rhizospheric bacterial community.

Biochemical profiling further demonstrated substantial functional variability. Catalase activity was observed in 80% of isolates, while most strains reduced nitrate. Approximately 60% were positive for Methyl Red and Voges-Proskauer tests, and ∼30% produced indole. Carbohydrate utilization patterns differentiated the isolates, with *Lysinibacillus macroides* exhibiting a broader assimilation spectrum than *Lysinibacillus fusiformis*, suggesting greater metabolic versatility and ecological adaptability (Table 1).

**TABLE 1:** Detail characterization of *Lysinibacillus macroides.*

S. No.	Experiment	*Lysinibacillus macroides*
1	Color	Cream
2	Texture	Smooth and opaque
3	Margin	Entire
4	Form	Circular
5	Gram staining	Gram-positive rods
6	Catalase	Positive
7	Methyl-Red (MR)	Negative
8	Voges-Proskauer (VP)	Positive
9	Indole	Negative
10	Nitrate reduction	Positive
11	Carbohydrate utilization	Glucose (+), Sucrose (+), Mannitol ( ± ), Lactose (-)
12	Amylase	Positive
13	Lipase	Negative
14	Protease	Positive
15	Urease	Positive
16	Cellulase	Variable ( ± )
17	Phosphate solubilization	+
18	Nitrogen fixation	Positive
19	Ammonia production	Positive
20	HCN production	Negative
21	IAA production	Positive
22	Siderophore production	Positive
23	Growth condition	Aerobic
24	Growth temperature	20–37°C

(+), Presence of trait; (-), Absence of trait.

### 3.2 Molecular identification

Representative high-efficiency isolates (2.B and 4.1) were subjected to 16S rRNA gene sequencing for molecular identification. BLAST analysis revealed > 99% sequence similarity of isolate 2.B with *Lysinibacillus macroides* and isolate 4.1 with *Lysinibacillus fusiformis*. The obtained sequences were deposited in GenBank under accession numbers PX482185 and PX482183, respectively.

### 3.3 Qualitative phosphate solubilization

Phosphate-solubilizing ability of the 23 rhizospheric isolates was assessed on Pikovskaya’s agar supplemented with tricalcium phosphate (TCP) as the insoluble phosphorus source. Clear halo formation surrounding bacterial colonies was considered indicative of TCP solubilization, and the Solubilization Index (SI) was calculated based on colony and halo diameters.

**FIGURE 1:**
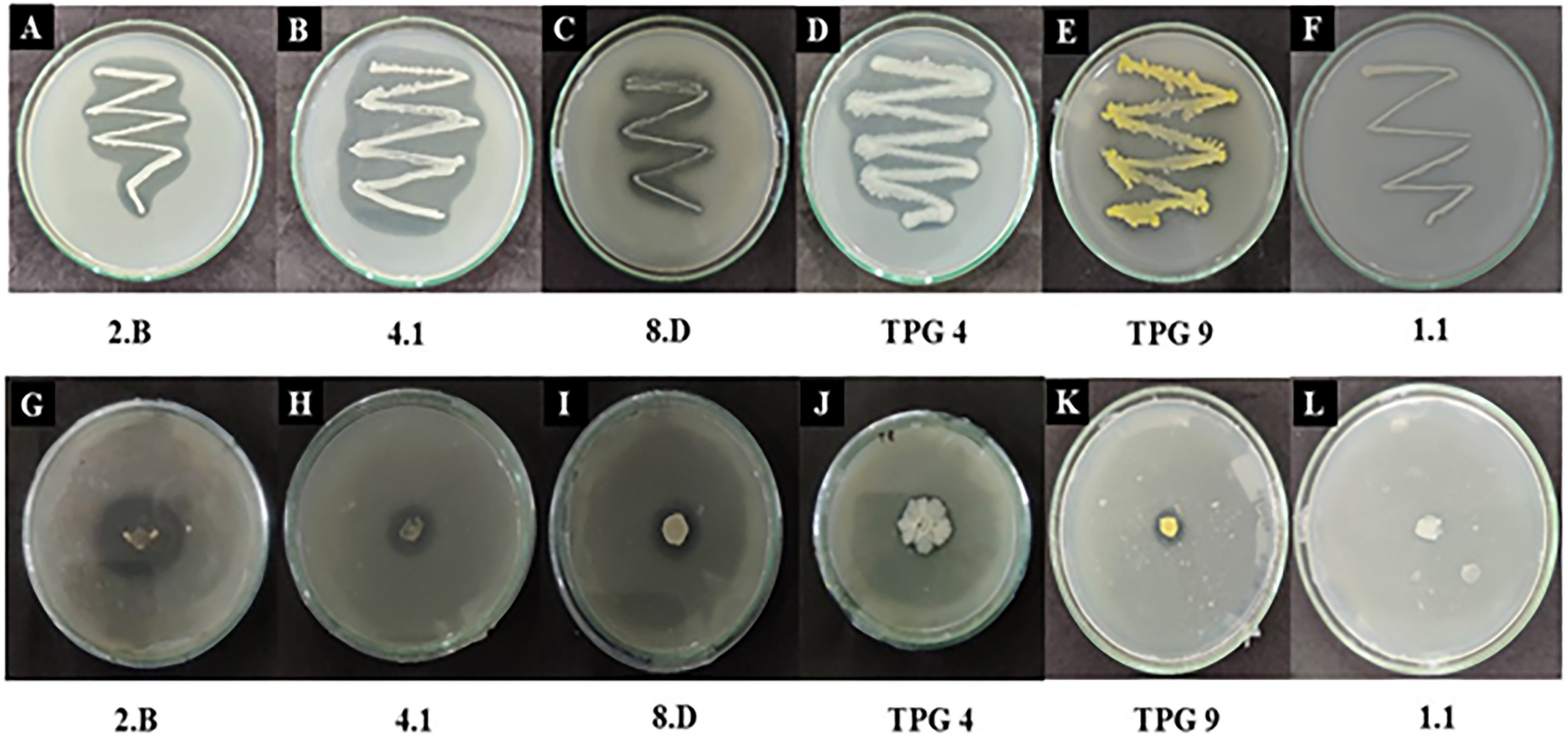
Qualitative phosphate solubilization by rhizobacterial isolates on Pikovskaya’s agar. **(A–F)** Represent streak inoculation and **(G–L)** represent spot inoculation assays. Clear halo zones surrounding colonies indicate tricalcium phosphate solubilization. Isolate 1.1 **(F,L)**, which showed no halo formation and was therefore considered negative.

Out of the 23 isolates screened, 17 exhibited measurable phosphate solubilization, while six isolates (1.1, 2.1, 8.B, 7.1, TPG7 and TPG8) showed no halo formation, indicating an inability to solubilize TCP under the tested conditions (Figure 1).

**FIGURE 2:**
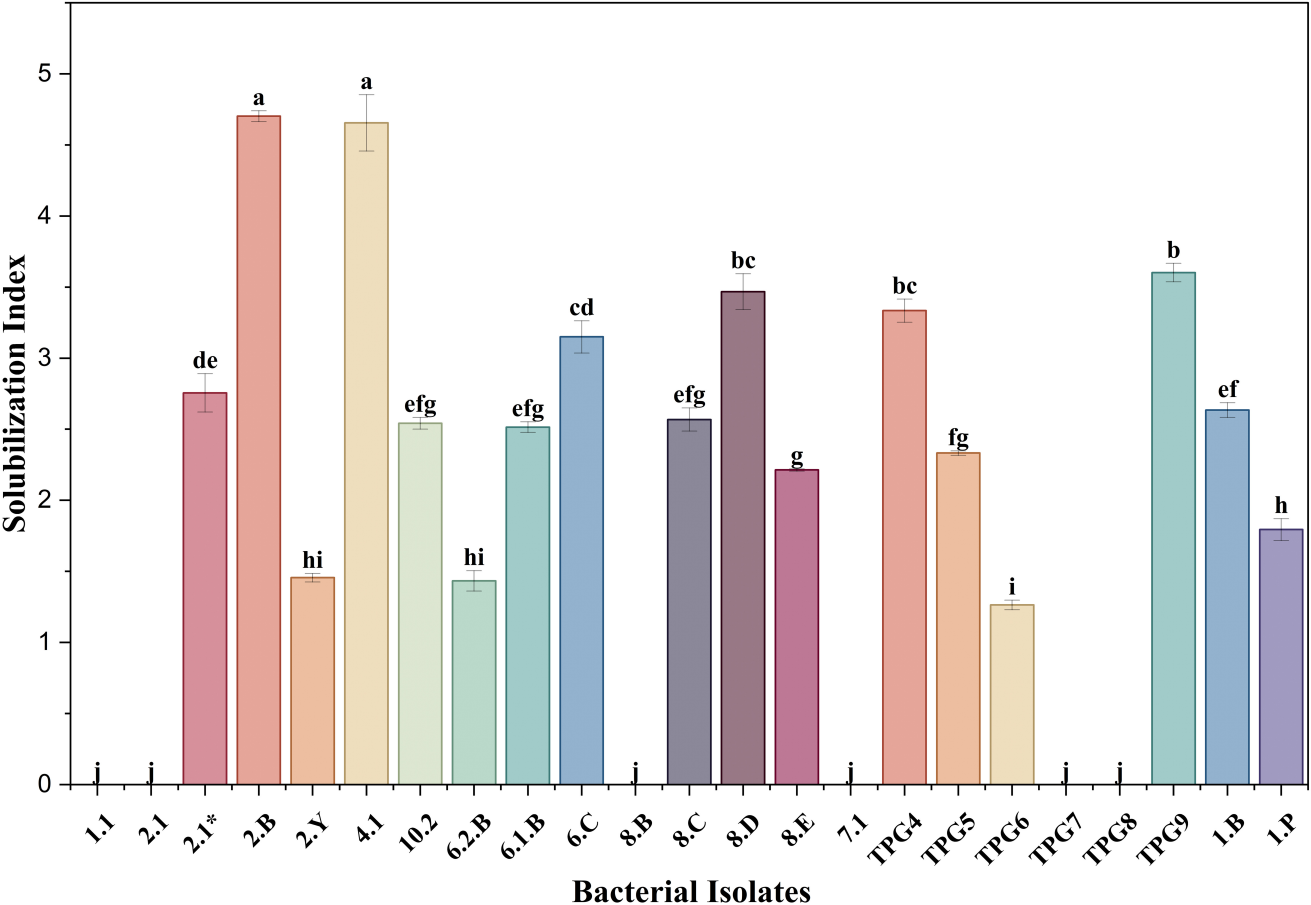
Solubilization index (SI) of rhizobacterial isolates on Pikovskaya’s agar. Values represent mean ± SD (*n* = 3). Different lowercase letters indicate significant differences (Tukey’s HSD, *p* ≤ 0.05). Isolates 2.B and 4.1 showed the highest solubilization, whereas several isolates exhibited negligible activity.

Significant variability in solubilization efficiency was observed among the positive isolates. The highest SI values were recorded for isolates 2.B (4.70 ± 0.06) and 4.1 (4.65 ± 0.34), followed by TPG9 (3.60 ± 0.11), 8.D (3.46 ± 0.21), TPG4 (3.33 ± 0.14) and 6.C (3.14 ± 0.19). Moderate solubilization activity was observed in isolates 2.1*, 10.2, 6.1.B, 8.C, TPG5 and 1.B. Lower but detectable activity was recorded for isolates 2.Y (1.45 ± 0.05), 6.2.B (1.43 ± 0.12), TPG6 (1.26 ± 0.05) and 1.P (1.79 ± 0.13) (Figure 2).

The marked differences in SI values among isolates indicate substantial functional heterogeneity in phosphorus-mobilizing capacity within the chili rhizosphere. Notably, isolates 2.B and 4.1 demonstrated superior halo formation with minimal standard deviation, suggesting stable and efficient TCP solubilization potential. These findings highlight the presence of highly active phosfate-solubilizing bacteria (PSB) within the rhizospheric microbiome, warranting further quantitative and *in-planta* validation.

### 3.4 Quantitative phosphate solubilization and pH dynamics under increasing TCP concentrations

**FIGURE 3:**
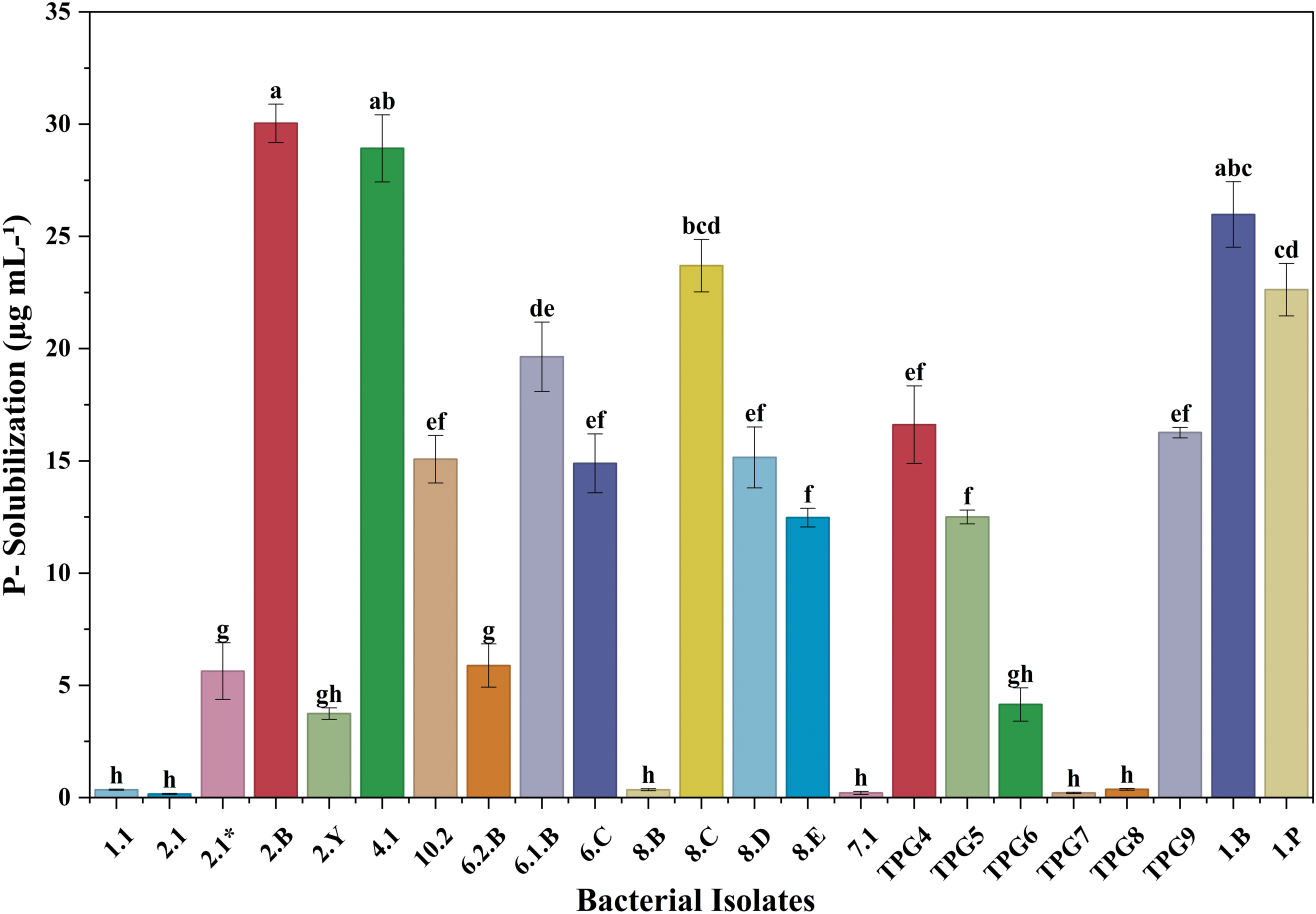
Soluble phosphate released by rhizobacterial isolates in NBRIP broth. Values are mean ± SD (*n* = 3). Different lowercase letters indicate significant differences (Tukey’s HSD, *p* ≤ 0.05), reflecting isolate-dependent variation in phosphate mobilization. The asterisk (*) denotes the isolate name.

Quantitative estimation of soluble phosphate in NBRIP broth revealed substantial variability among the rhizobacterial isolates, with concentrations ranging from negligible levels (0.16–0.36 μg mL^–1^) to a maximum of 30.03 ± 1.48 μg mL^–1^. Isolate 2.B exhibited the highest phosphate release (30.03 ± 1.48 μg mL^–1^), closely followed by 4.1 (28.92 ± 2.58 μg mL^–1^). Elevated soluble phosphorus levels were also recorded in 1.B (25.97 ± 2.52 μg mL^–1^), 8.C (23.69 ± 2.02 μg mL^–1^) and 1.P (22.62 ± 2.01 μg mL^–1^). Moderate phosphate mobilization was observed in 6.1.B, TPG4, TPG9, 8.D, 10.2 and 6.C, whereas several isolates exhibited minimal solubilization ( < 1 μg mL^–1^) (Figure 3).

The final pH of the culture filtrates ranged from 3.1 to 4.1, indicating pronounced acidification relative to the initial neutral pH (7.0). Highly efficient solubilizers such as 2.B (pH 3.2) and 4.1 (pH 3.3) demonstrated marked medium acidification, supporting the involvement of organic acid production in tricalcium phosphate (TCP) dissolution. In contrast, weak solubilizers maintained comparatively higher pH values (3.9–4.1), reflecting limited proton release and reduced mineral phosphate mobilization. To further elucidate substrate-dependent pH modulation, isolates were evaluated under graded TCP concentrations (0.05–0.40 mg mL^–1^). A general trend of progressive pH reduction with increasing TCP concentration was observed in several efficient isolates. Notably, 2.B exhibited a gradual decline from pH 4.8 (0.05 mg mL^–1^) to 3.2 (0.40 mg mL^–1^), indicating enhanced acidification under elevated phosphate load. Similarly, 1.1 showed a consistent decrease from pH 3.6 to 3.2, while 2.1 declined from 3.7 to 3.1 across the concentration gradient (Table 2). Isolate 4.1 demonstrated moderate but sustained acidification (pH 4.3 at 0.05 mg mL^–1^ to 3.3 at 0.40 mg mL^–1^), suggesting stable metabolic adaptation under increasing substrate levels. In contrast, certain isolates such as 2.Y, 6.2.B, 8.C, and 6.1.B displayed fluctuating pH responses, indicating differential regulatory mechanisms or buffering interactions at higher TCP concentrations.

**TABLE 2:** Effect of increasing tricalcium phosphate (TCP) concentrations on culture pH of rhizobacterial isolates in NBRIP broth.

Isolate	0.05 mg/mL	0.10 mg/mL	0.20 mg/mL	0.30 mg/mL	0.40 mg/mL
1.1	3.6	3.6	3.5	3.3	3.2
2.1	3.7	3.5	3.3	3.1	3.1
2.1*	4.1	3.9	3.9	3.9	3.8
2.B	4.8	4.5	3.9	3.4	3.2
2.Y	3.9	3.3	3.9	4.0	3.9
4.1	4.3	3.3	3.6	3.4	3.3
10.2	4.2	4.1	4.1	3.9	3.9
6.2.B	4.1	4.2	4.5	3.9	4.0
6.1.B	4.4	3.8	4.1	3.6	4.1
6.C	4.1	4.2	4.2	4.0	3.9
8.B	4.3	4.1	4.0	3.7	4.0
8.C	3.8	4.6	3.9	3.7	3.8
8.D	4.5	4.1	3.9	4.0	4.0
8.E	4.3	4.4	4.2	4.0	3.9
7.1	3.9	4.1	3.9	4.0	4.0
TPG4	4.1	4.2	4.0	4.0	3.8
TPG5	4.1	3.8	4.1	3.7	3.8
TPG6	3.8	4.1	3.9	3.8	3.7
TPG7	4.3	3.9	4.0	3.8	4.0
TPG8	4.0	4.2	3.9	4.0	3.9
TPG9	4.4	4.1	3.9	3.8	3.7
1.B	4.3	4.2	3.8	3.9	3.8
1.P	3.7	3.9	4.1	4.0	3.8

Decreased pH indicates acidification-mediated phosphate solubilization. The asterisk (*) denotes the isolate name.

Overall, the data indicate a concentration-responsive acidification pattern in efficient phosphate solubilizers, particularly 2.B and 4.1. The progressive pH decline with increasing TCP concentration likely reflects intensified organic acid secretion to mobilize higher mineral phosphate levels. These findings substantiate the mechanistic link between acid production and phosphate solubilization and reinforce the robustness of selected isolates for application in phosphorus-deficient agroecosystems (Table 3). This integrated approach enabled the quantitative assessment of phosphate solubilization capacity under standard conditions as well as under increasing TCP concentrations, thereby facilitating evaluation of substrate-dependent solubilization efficiency.

**TABLE 3:** Amount of phosphate solubilized (μgmL^–1^) on solid and liquid media and pH change by PSB isolates from chili rhizosphere.

S. no.	Bacterial isolates	Solubilization index (SI) (mean ± SD)	P- Solubilization (μ g mL^−1^) (mean ± SD)	pH
1	1.1	0	0.34 ± 0.04	3.2
2	2.1	0	0.16 ± 0.04	3.1
3	2.1*	2.75 ± 0.23	5.64 ± 2.19	3.8
4	2.B	4.70 ± 0.06	30.03 ± 1.48	3.2
5	2.Y	1.45 ± 0.05	3.74 ± 0.44	3.9
6	4.1	4.65 ± 0.34	28.92 ± 2.58	3.3
7	10.2	2.54 ± 0.07	15.07 ± 1.83	3.9
8	6.2.B	1.43 ± 0.12	5.88 ± 1.67	4.0
9	6.1.B	2.51 ± 0.06	19.63 ± 2.67	4.1
10	6.C	3.14 ± 0.19	14.89 ± 2.27	3.9
11	8.B	0	0.34 ± 0.08	4.0
12	8.C	2.56 ± 0.14	23.69 ± 2.02	3.8
13	8.D	3.46 ± 0.21	15.15 ± 2.34	4.0
14	8.E	2.21 ± 0.01	12.47 ± 0.72	3.9
15	7.1	0	0.20 ± 0.12	4.0
16	TPG4	3.33 ± 0.14	16.61 ± 2.99	3.8
17	TPG5	2.33 ± 0.02	12.05 ± 0.52	3.8
18	TPG6	1.26 ± 0.05	4.14 ± 1.29	3.7
19	TPG7	0	0.20 ± 0.04	4.0
20	TPG8	0	0.36 ± 0.07	3.9
21	TPG9	3.60 ± 0.11	16.25 ± 0.40	3.7
22	1.B	2.63 ± 0.08	25.97 ± 2.52	3.8
23	1.P	1.79 ± 0.13	22.62 ± 2.01	3.8

The asterisk (*) denotes the isolate name.

### 3.5 Correlation between phosphate solubilization parameters and pH

**FIGURE 4:**
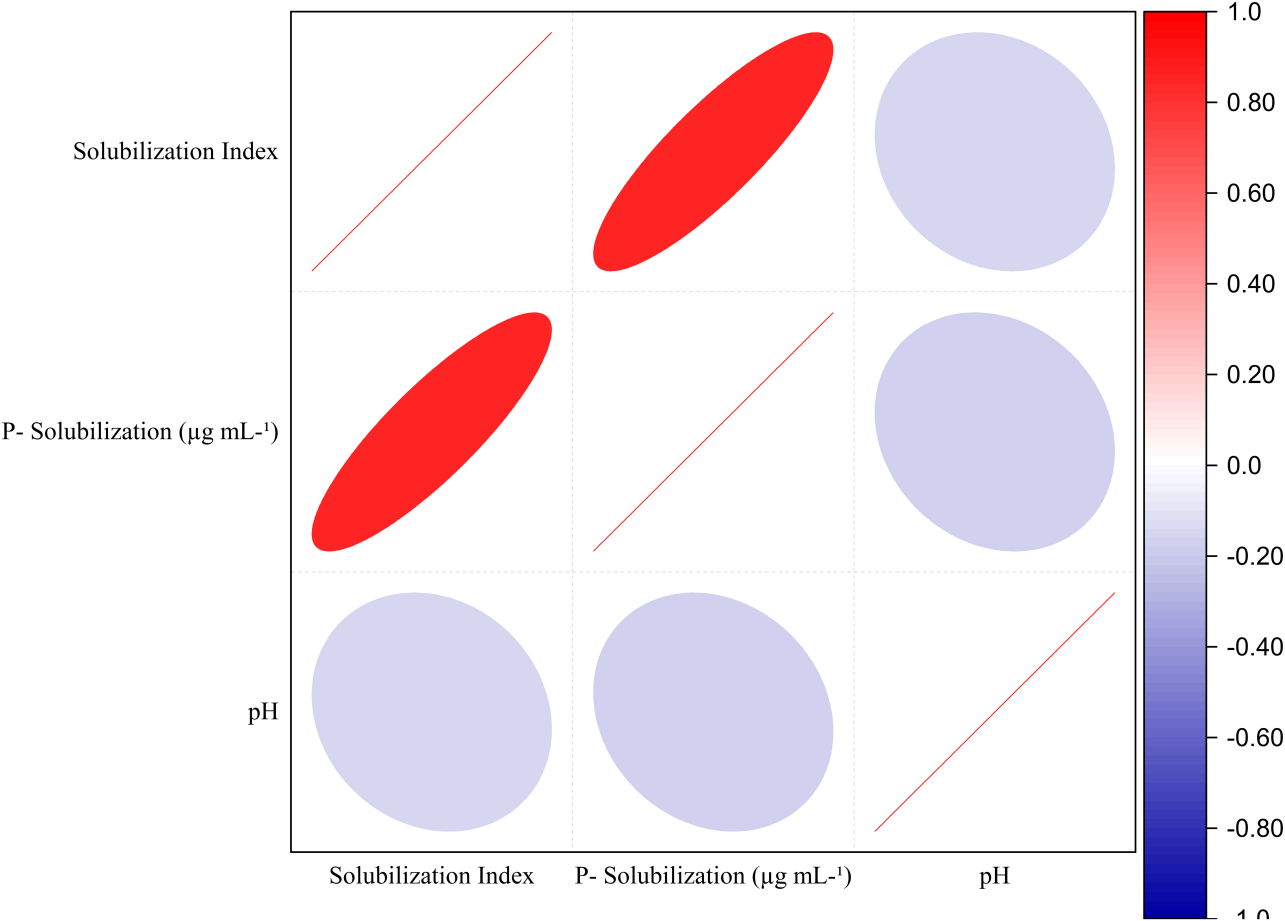
Pearson correlation matrix among solubilization index (SI), soluble phosphate concentration (μg mL^–1^), and final culture pH. Ellipse orientation and color intensity represent the strength and direction of correlations, with red indicating positive and blue indicating negative associations. Strong positive correlation was observed between SI and soluble phosphate, while both parameters showed negative correlation with pH, indicating acidification-driven phosphate solubilization.

**FIGURE 5:**
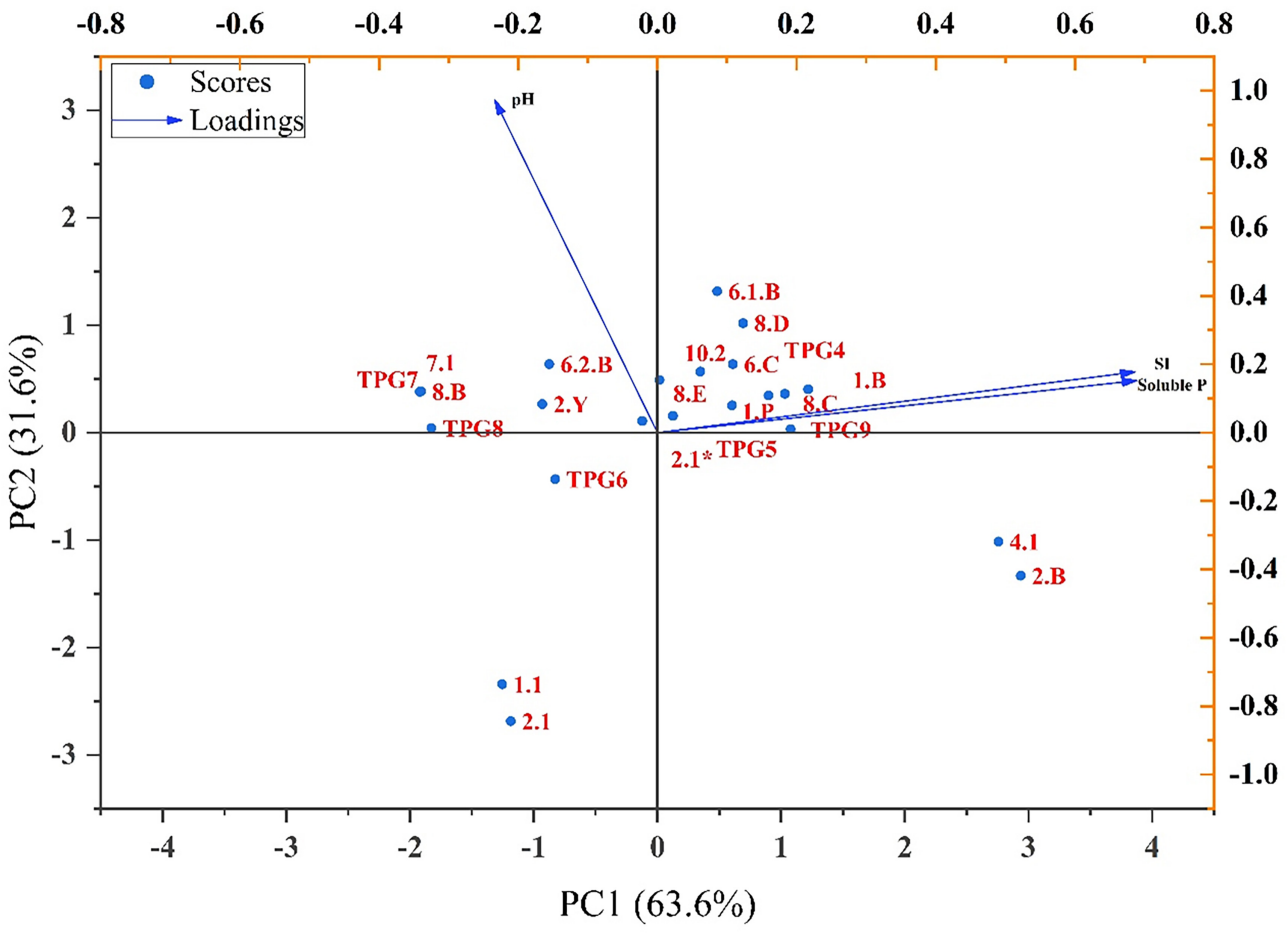
Principal component analysis (PCA) biplot showing the distribution of isolates (scores) and associated variables (loadings). PC1 and PC2 explain 63.6 and 31.6% of the total variance, respectively. Blue points represent individual isolates, while arrows indicate variable loadings (pH, Soluble P, and SI). The direction and length of vectors reflect the strength and influence of variables on sample clustering. Positive correlation is indicated by vectors pointing in the same direction, while opposite directions indicate negative correlation.

Pearson’s correlation analysis demonstrated a strong positive relationship between Solubilization Index (SI) and soluble phosphate concentration (*r* = 0.86, *p* < 0.01), indicating that halo zone formation on solid medium reliably reflects quantitative phosphate release in broth culture. A significant negative correlation was observed between soluble phosphate and final culture pH (*r* = −0.91, *p* < 0.01), while SI showed a weak to moderate inverse association with pH (*r* ≈−0.14) (Figure 4). These results indicate that enhanced phosphate mobilization was consistently accompanied by medium acidification. The strong inverse relationship between soluble phosphate concentration and pH supports acidification-driven solubilization, likely mediated by organic acid production and proton extrusion, facilitating tricalcium phosphate dissolution through Ca^2 +^ chelation and orthophosphate release. To further resolve the multivariate relationships among these parameters, Principal Component Analysis (PCA) was performed. The first two principal components explained a substantial proportion of total variance (PC1 = 63.6% and PC2 = 31.6%; cumulative = 95.2%), indicating a robust representation of the dataset (Figure 5). PC1 was predominantly associated with soluble phosphate and SI, both of which exhibited strong positive loadings, confirming their close functional linkage. In contrast, pH was negatively associated along this axis, reinforcing its inverse relationship with phosphate solubilization efficiency. PC2 was largely driven by pH variation, reflecting differences in acidification dynamics among isolates. The PCA biplot distinctly differentiated the isolates according to their functional performance. Isolates positioned along the positive PC1 axis, particularly 2.B and 4.1, showed a strong association with elevated soluble phosphate release and higher solubilization index (SI). In contrast, isolates clustered along the negative PC1 axis demonstrated comparatively lower phosphate-solubilizing potential. Furthermore, the opposing orientation of the pH vector relative to soluble phosphate and SI vectors substantiates acidification as the central mechanism governing phosphate mobilization.

Collectively, both univariate (correlation) and multivariate (PCA) analyses converge to demonstrate that phosphate solubilization is tightly coupled with pH reduction. The clustering of high-performing isolates (2.B and 4.1) along the direction of phosphate-related variables further highlights their superior functional efficiency, reinforcing their potential as effective biofertilizer candidates for sustainable phosphorus management.

### 3.6 Abiotic stress tolerance and climate resilience of *Lysinibacillus macroides*

**FIGURE 6:**
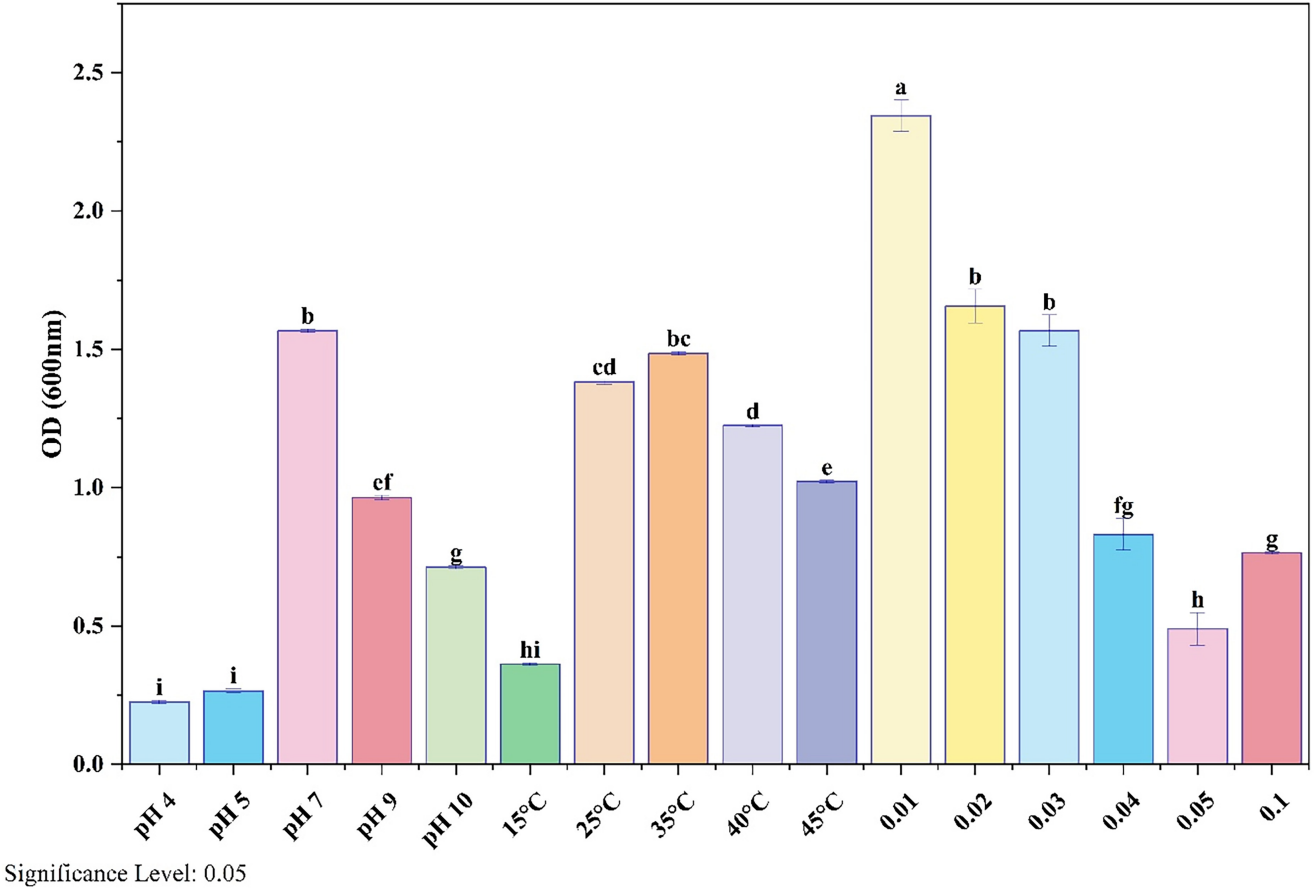
Growth performance of *Lysinibacillus macroides* expressed as mean optical density (OD_600_) ± SD; *n* = 3) under varying pH (4–10), temperature (15–45°C), and NaCl concentrations (0.01–0.1%). Error bars indicate standard deviation, reflecting variability among replicates. The different letters are bar labels indicating statistically significant differences among treatments at *p* < 0.05.

*Lysinibacillus macroides* exhibited pronounced climate-resilient characteristics, as demonstrated by its ability to sustain growth across a wide range of abiotic stress conditions, including pH (4–10), temperature (15–45°C), and salinity (0.01–0.1% NaCl), with statistically significant variations among treatments (*p* ≤ 0.05). The isolate showed a clear optimum at neutral pH, with maximum growth recorded at pH 7 (1.57 ± 0.01), while still maintaining measurable growth under both acidic (pH 4: 0.23 ± 0.01; pH 5: 0.27 ± 0.01) and alkaline conditions (pH 9: 0.96 ± 0.01; pH 10: 0.71 ± 0.01), indicating broad pH adaptability. Temperature response analysis further revealed a mesophilic optimum at 35°C (1.40 ± 0.01), closely followed by 25°C (1.38 ± 0.01), with sustained growth even at elevated temperatures of 40°C (1.22 ± 0.02) and 45°C (1.02 ± 0.01), highlighting its thermotolerant nature. Although growth was comparatively lower at 15°C (0.36 ± 0.02), the persistence of viable biomass suggests partial adaptation to low-temperature stress. Salinity stress profiling indicated that the isolate performs optimally under low osmotic conditions, with maximum growth at 0.01% NaCl (2.34 ± 0.09), followed by a gradual decline with increasing salt concentration (0.02%: 1.66 ± 0.10; 0.03%: 1.57 ± 0.09). Despite this reduction, the bacterium retained appreciable growth at moderate salinity levels and even exhibited measurable survival at higher concentrations (0.05%: 0.49 ± 0.10; 0.1%: 0.76 ± 0.02), suggesting the activation of adaptive osmotic regulation mechanisms (Figure 6).

*Lysinibacillus macroides* exhibits high physiological plasticity by tolerating variations in pH, temperature, and salinity, underscoring its climate-resilient potential for application in stress-prone agroecosystems.

### 3.7 Effect of rhizobacterial seed biopriming on germination and early seedling growth

**FIGURE 7:**
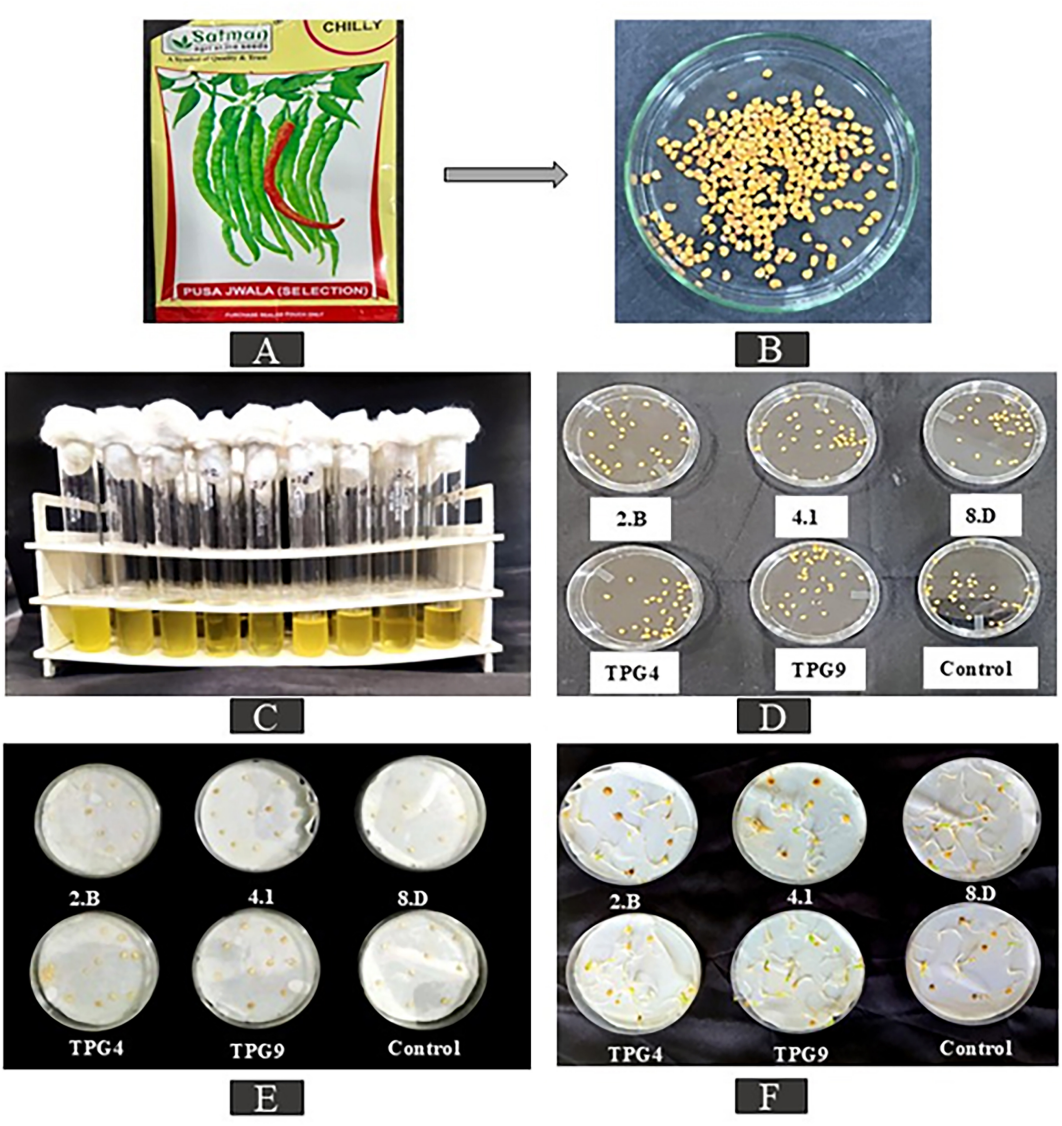
Seed biopriming assay of selected rhizobacterial isolates. **(A)** Chili Seed: PUSA JWALA. **(B)** Surface-sterilized chili seeds. **(C)** Liquid culture of Bacterial isolates. **(D)** Surface-sterilized chili seeds soaked in bacterial suspension. **(E)** Germination setup on sterile moist filter paper. **(F)** Seedling emergence after incubation.

Seed biopriming with rhizobacterial isolates significantly influenced germination performance and early seedling development of chili (*Capsicum annuum* L.) compared to the uninoculated control. Germination percentage varied markedly among treatments, ranging from 53.33 to 96.66%, indicating pronounced isolate-dependent variability. Isolate 2.B exhibited the highest germination (96.66%), followed by 4.1 (93.33%), whereas 8.D, TPG4, and 1.B (86.66%) also demonstrated substantial improvement relative to the control (50%). In contrast, isolates such as TPG6 and 7.1 (56.66%) showed comparatively moderate responses. These differences suggest variability in the ability of individual isolates to stimulate early physiological processes associated with radicle emergence and metabolic activation during germination (Figure 7).

**FIGURE 8:**
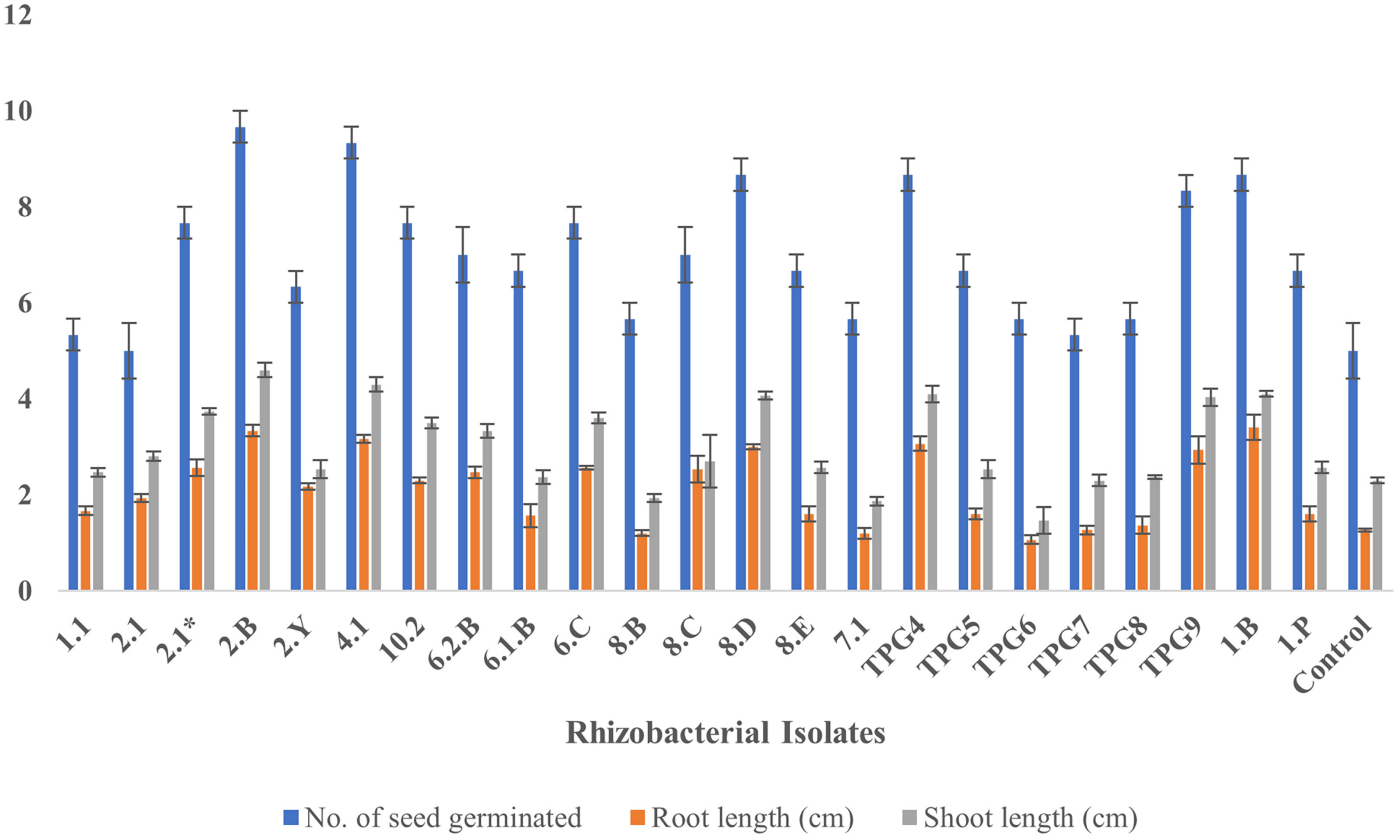
Effect of rhizobacterial seed biopriming on germination and early seedling growth of chili. Bars represent mean ± SD (*n* = 3) for number of seeds germinated, root length (cm), and shoot length (cm). Variability among isolates indicates differential influence on germination efficiency and seedling development compared to the uninoculated control. The asterisk (*) denotes the isolate name.

**FIGURE 9:**
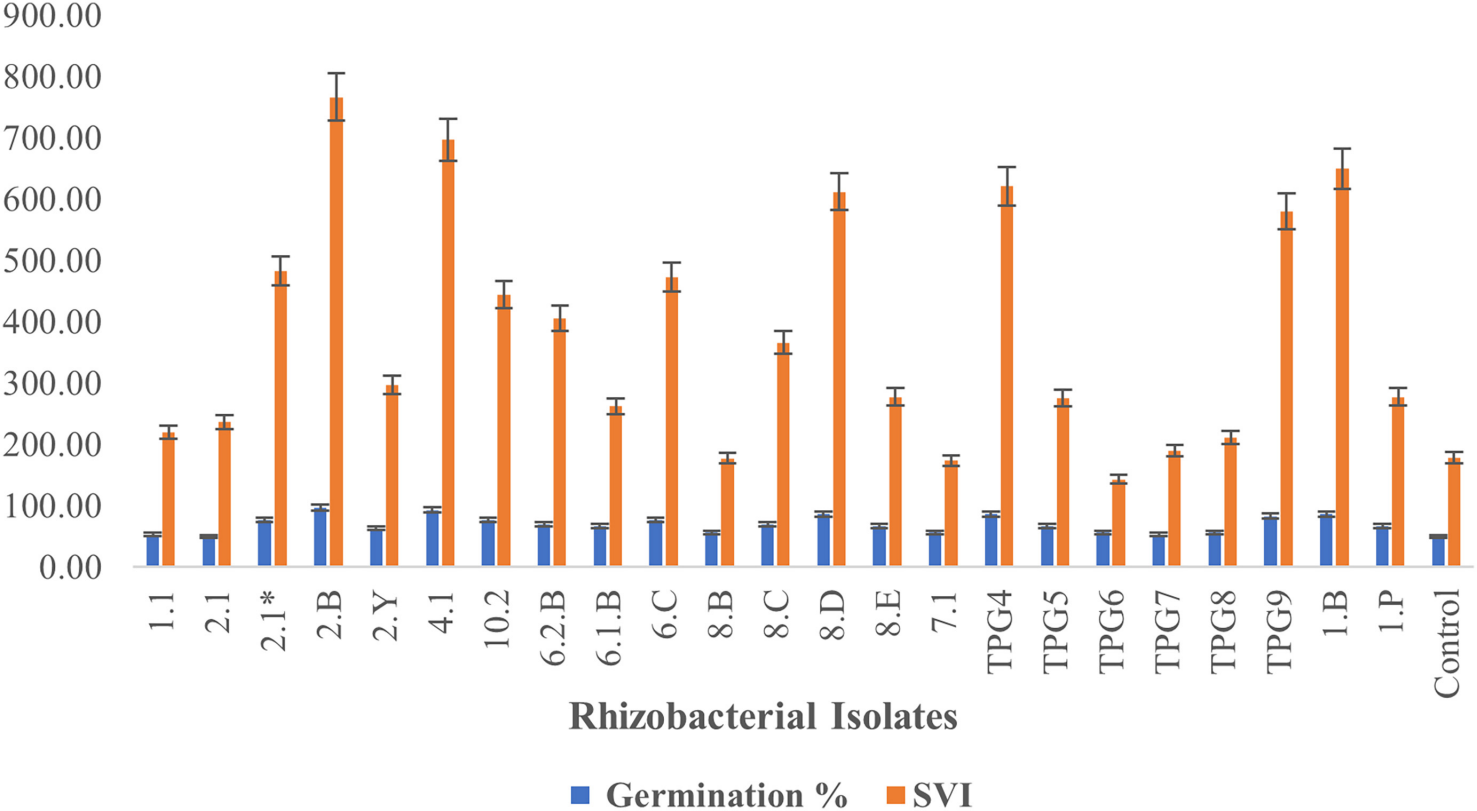
Effect of rhizobacterial biopriming on germination percentage and Seedling Vigor Index (SVI) of chili seeds. Bars represent mean values of three biological replicates (*n* = 3), and error bars indicate standard deviation (SD). Differences among isolates indicate variable effects on seed germination and early seedling growth relative to the uninoculated control. The asterisk (*) denotes the isolate name.

Significant treatment effects were also observed for root and shoot elongation (*p* ≤ 0.05). Mean root length ranged from 1.07 cm (TPG6) to 3.33 cm (2.B), while shoot length varied from 1.47 to 4.60 cm across treatments. Isolate 2.B consistently recorded the greatest enhancement in both root (3.33 cm) and shoot length (4.60 cm), followed closely by 4.1 and 1.B, indicating strong stimulation of early seedling growth. Additionally, isolates 8.D, TPG4, and TPG9 exhibited notable elongation responses. In comparison, control seedlings showed reduced growth (mean root length ∼ 1.27 cm; shoot length ∼ 2.30 cm), confirming the positive influence of bacterial inoculation on vegetative development (Figure 8). The cumulative improvement in germination and seedling growth was reflected in significant variation in the Seedling Vigor Index (SVI). SVI values ranged from 143.55 to 766.88, with the highest recorded in 2.B (766.88), followed by 4.1 (696.88) and 1.B (650.00). Several additional isolates, including TPG4 (621.11), 8.D (612.44), and TPG9 (580.55), also demonstrated strong vigor enhancement (Figure 9). In contrast, the control exhibited a substantially lower SVI (178.33), reinforcing the effectiveness of rhizobacterial biopriming (Table 4). One-way ANOVA confirmed significant differences among treatments for germination percentage, root length, shoot length, and SVI (*p* ≤ 0.05), and Tukey’s HSD test indicated that isolates 2.B and 4.1 formed a statistically superior group for most growth parameters. Collectively, these findings demonstrate that rhizobacterial biopriming markedly enhances early germination and seedling vigor in chili, with isolates 2.B and 4.1 emerging as the most promising candidates for further functional validation and bioinoculant development.

**TABLE 4:** Germination percentage and seedling vigor index (SVI) of chili seeds following biopriming with rhizobacterial isolates.

S. no.	Bacterial isolates	Germination %	SVI
1	1.1	53.33	220.44
2	2.1	50	236.66
3	2.1*	76.66	483
4	2.B	96.66	766.88
5	2.Y	63.33	297.66
6	4.1	93.33	696.88
7	10.2	76.66	444.66
8	6.2.B	70	406
9	6.1.B	66.66	262.22
10	6.C	76.66	472.77
11	8.B	56.66	177.55
12	8.C	70	366.33
13	8.D	86.66	612.44
14	8.E	66.66	277.77
15	7.1	56.66	173.77
16	TPG4	86.66	621.11
17	TPG5	66.66	275.55
18	TPG6	56.66	143.55
19	TPG7	53.33	190.22
20	TPG8	56.66	211.55
21	TPG9	83.33	580.55
22	1.B	86.66	650
23	1.P	66.66	277.77
24	Control	50	178.33

Values represent mean of three replicates; control denotes uninoculated seeds. The asterisk (*) denotes the isolate name.

## 4 Discussion

The present study provides comprehensive evidence that phosphate solubilization by chili rhizospheric isolates is predominantly governed by acidification-driven mineral dissolution, with strong quantitative support from both solid and liquid assays. The significant positive correlation between Solubilization Index (SI) and soluble phosphate concentration (*r* = 0.86, *p* < 0.01), coupled with the pronounced negative correlation between soluble phosphate and final culture pH (*r* = −0.91, *p* < 0.01), confirms that halo formation on Pikovskaya’s agar is a reliable proxy for phosphate mobilization in liquid systems. These findings are consistent with earlier reports in classical phosphate-solubilizing genera such as *Bacillus* and *Pseudomonas*, where organic acid-mediated acidification plays a central role in tricalcium phosphate (TCP) dissolution (Khan et al., 2023). Recent studies have further substantiated this mechanism, demonstrating similar strong correlations between pH decline and phosphate release in PSB such as *Bacillus subtilis* and *Pseudomonas fluorescens*, where soluble phosphate levels exceeding 25–35 μg mL^–1^ were associated with culture pH reductions below 4.5 (Bakki et al., 2024). Importantly, the present study extends the currently established mechanistic understanding of phosphate solubilization to the comparatively underexplored genus *Lysinibacillus*, thereby broadening the functional and taxonomic diversity of recognized phosphate-solubilizing microorganisms. Previous studies have predominantly focused on conventional genera such as *Bacillus*, *Pseudomonas*, and other Gram-negative bacteria, with comparatively limited information available on the phosphate-solubilizing mechanisms and agronomic relevance of spore-forming *Lysinibacillus* spp. In this context, the present study not only demonstrates efficient phosphate solubilization in a Gram-positive, spore-forming genus but also integrates mechanistic interpretation with plant-based validation, thereby addressing an important knowledge gap regarding the functional potential and ecological relevance of resilient phosphate-solubilizing bacteria for biofertilizer development.

At the mechanistic level, phosphate solubilization in efficient isolates (particularly 2.B and 4.1) is most plausibly mediated through the secretion of low-molecular-weight organic acids, which facilitate mineral dissolution via dual mechanisms of proton extrusion and cation chelation. Organic acids such as gluconic, citric, and oxalic acids are known to lower rhizospheric pH and chelate Ca^2 +^ ions bound to phosphate complexes, thereby releasing soluble orthophosphate (H_2_PO_4_^–^/HPO_4_^2–^) (Luo et al., 2025). In many phosphate-solubilizing bacteria, this process is biochemically linked to the direct oxidation pathway of glucose, catalyzed by membrane-bound glucose dehydrogenase (GCD) in association with the cofactor pyrroloquinoline quinone (PQQ), resulting in gluconic acid production (García-Contreras et al., 2026). Recent molecular studies have confirmed the involvement of gcd and pqq gene clusters in high-efficiency PSB strains, with enhanced expression correlating with increased gluconic acid production and phosphate solubilization efficiency (Chen et al., 2024). Although gene-level validation was beyond the scope of the present study, the strong inverse relationship between pH and soluble phosphate strongly supports the involvement of such metabolic pathways in *Lysinibacillus macroides*. Furthermore, the observed substrate-dependent acidification under increasing TCP concentrations suggests a metabolically regulated response rather than passive acid diffusion. The progressive decline in pH in isolate 2.B (from 4.8 to 3.2) with increasing phosphate load indicates inducible metabolic plasticity, likely driven by enhanced carbon flux through organic acid biosynthesis pathways. Comparable substrate-responsive acidification patterns have been reported in recent studies on *Bacillus megaterium* and *Enterobacter cloacae*, where increased TCP availability stimulated organic acid secretion and enhanced phosphate mobilization (Sokolova et al., 2025). Such adaptive responses have been associated with improved phosphate mobilization efficiency under nutrient-limited conditions and are considered critical for functional performance in heterogeneous soil environments (Bucio et al., 2025).

Marked inter-strain variability observed across qualitative and quantitative assays underscores the functional heterogeneity of the rhizospheric microbiome. While 17 out of 23 isolates demonstrated measurable phosphate solubilization, only a subset exhibited high efficiency, with isolates 2.B and 4.1 showing SI values exceeding 4.6 and soluble phosphate concentrations above 28 μg mL^–1^. This observation reinforces the concept that phosphate solubilization is a strain-specific trait rather than a genus-level characteristic, governed by differences in metabolic regulation, carbon utilization efficiency, and organic acid production pathways (Amri et al., 2023). Recent comparative studies have similarly reported substantial intra-genus variability among PSB isolates, with only 20–30% of screened strains demonstrating high-efficiency solubilization, emphasizing the importance of strain-level selection (Kaur and Kaur, 2020). Notably, discrepancies between solid and liquid assays observed in certain isolates further highlight the limitations of relying solely on plate-based screening methods. Halo zone formation may overestimate functional capacity due to diffusion effects and localized acidification, whereas liquid assays provide a more accurate representation of phosphate mobilization dynamics. This observation is consistent with recent methodological studies recommending the combined use of plate and broth assays to improve reliability in PSB screening (Bansal et al., 2025). The integration of both approaches in the present study therefore strengthens the robustness of strain selection and aligns with recent methodological recommendations in PGPR research.

The functional relevance of phosphate solubilization was substantiated through seed biopriming assays, wherein isolates 2.B and 4.1 significantly enhanced germination percentage and Seedling Vigor Index. This concordance between biochemical activity and plant growth promotion suggests a direct mechanistic linkage between phosphorus mobilization and early plant development. Phosphorus is a critical component of ATP, nucleic acids, and membrane phospholipids, and its availability during germination directly influences energy metabolism, cell division, and radicle emergence (Stigter and Plaxton, 2015). Enhanced root elongation observed in treated seedlings likely reflects improved phosphorus acquisition, which in turn facilitates nutrient uptake and early biomass accumulation (Cai et al., 2023). Comparable improvements in germination and seedling vigor following PSB-mediated seed biopriming have been reported in crops such as wheat, maize, and chili, further supporting the role of phosphate-solubilizing bacteria in enhancing early stage plant development (Shil et al., 2025). Beyond phosphorus mobilization, the multifunctional plant growth-promoting attributes of *Lysinibacillus macroides* including indole-3-acetic acid (IAA) production, siderophore secretion, and nitrogen metabolism suggest a synergistic mode of action that extends beyond nutrient solubilization. Such multifunctionality is increasingly recognized as a critical determinant of biofertilizer efficacy, as it enables simultaneous modulation of plant hormonal balance, nutrient acquisition, and stress tolerance (Gayithri et al., 2026). From an ecological and agronomic perspective, the spore-forming nature of *Lysinibacillus* confers significant advantages for field application. Endospore formation enhances survival under abiotic stress conditions such as desiccation, temperature fluctuations, and nutrient limitation, which are prevalent in semi-arid agroecosystems (Harishchandra et al., 2025). Recent studies have highlighted the superior field persistence and formulation stability of spore-forming PSB compared to non-sporulating strains, reinforcing their suitability for biofertilizer development (Samantaray et al., 2024). The ability of *Lysinibacillus macroides* to sustain substantial growth across a wide pH spectrum, elevated temperatures (up to 45°C), and varying salinity levels confirms its robustness under multiple abiotic stresses, a hallmark of climate-resilient PGPR. This trait improves formulation stability, shelf life, and persistence in soil, thereby addressing key limitations associated with non-sporulating PGPR (Soares et al., 2023).

## 5 Conclusion

The present investigation establishes *Lysinibacillus macroides* as a functionally competent and agronomically relevant phosphate-solubilizing bacterium within the chili rhizosphere. Through an integrated evaluation encompassing qualitative halo formation, quantitative phosphate release, pH dynamics under graded substrate concentrations, correlation analysis, and plant-based validation, the study provides strong evidence that mineral phosphate mobilization in selected isolates is predominantly mediated by acidification-driven dissolution of tricalcium phosphate. The observed inverse relationship between soluble phosphorus and culture pH supports a metabolically regulated mechanism involving proton extrusion and organic acid-mediated chelation. Phosphate solubilization was found to be strongly strain-dependent, emphasizing the necessity of isolate-level screening for effective biofertilizer development. Among the tested isolates, 2.B (*L. macroides*) and 4.1 (*L. fusiformis*) consistently demonstrated superior performance across *in vitro* assays and seed biopriming experiments. Their ability to enhance germination, root elongation, and Seedling Vigor Index confirms that phosphate mobilization translated into measurable improvements in early plant growth. This functional validation highlights the importance of phosphorus availability during early developmental stages. Beyond phosphorus mobilization, the multifunctional plant growth-promoting traits of *L. macroides* including IAA production, siderophore synthesis, and nitrogen metabolism along with its spore-forming ability, enhance its ecological adaptability, formulation stability, and persistence under fluctuating environmental conditions. *Lysinibacillus macroides* demonstrated strong climate resilience by sustaining growth across diverse pH, temperature, and salinity conditions, highlighting its potential for use in stress-prone agroecosystems. These attributes support its practical applicability as a bioinoculant for improving nutrient use efficiency in phosphorus-limited soils, particularly in semi-arid agroecosystems.

By expanding the taxonomic diversity of validated phosphate-solubilizing microorganisms, this study contributes to the development of biologically sustainable alternatives to chemical fertilizers. The identified strains hold promise for integration into biofertilizer formulations aimed at reducing fertilizer dependency and improving crop productivity. Future studies focusing on field validation, formulation optimization, and molecular characterization will be essential to confirm large-scale applicability.

## Data Availability

The raw data supporting the conclusions of this article will be made available by the authors, without undue reservation.
